# Nanoarchitectonics to entrap living cells in silica-based systems: encapsulations with yolk–shell and sepiolite nanomaterials

**DOI:** 10.3762/bjnano.14.43

**Published:** 2023-04-25

**Authors:** Celia Martín-Morales, Jorge Fernández-Méndez, Pilar Aranda, Eduardo Ruiz-Hitzky

**Affiliations:** 1 Materials Science Institute of Madrid, CSIC, C/ Sor Juana Inés de la Cruz 3, 28049 Madrid, Spain; 2 Faculty of Science, Autonomous University of Madrid (UAM), C/ Francisco Tomás y Valiente 7, 28049 Madrid, Spain; 3 Faculty of Biological Sciences, Complutense University of Madrid (UCM), C/ José Antonio Novais 12, 28040 Madrid, Spain

**Keywords:** biohybrids, cell immobilization, encapsulation, microorganism entrapment, silicates

## Abstract

In the present work, the bottom-up fabrication of biohybrid materials using a nanoarchitectonics approach has been applied to entrap living cells. Unicellular microorganisms, that is, cyanobacteria and yeast cells, have been immobilized in silica and silicate-based substrates organized as nanostructured materials. In a first attempt, matrices based on bionanocomposites of chitosan and alginate incorporating sepiolite clay mineral and shaped as films, beads, or foams have been explored for the immobilization of cyanobacteria. It has been observed that this type of biohybrid substrates leads to serious problems regarding the long-time survival of the encapsulated microorganisms. Alternative procedures using silica-based matrices with low sodium content, generated by sol–gel methods, as well as pre-synthesised yolk–shell bionanohybrids have been studied subsequently. Optical microscopy and SEM confirm that the silica shell microstructures provide a reduced contact between cells. The inorganic matrix increases the survival of the cells and maintains their bioactivity. Thus, the encapsulation efficiency is improved compared to the approach using a direct contact of cells in a silica matrix. Encapsulated yeast produced ethanol over a period of several days, pointing out the useful biocatalytic potential of the approach and suggesting further optimization of the present protocols.

## Introduction

Bio-inorganic hybrid nanomaterials with highly specific functionalities can be prepared following Nature’s design approaches [1]. A wide range of materials resulting from the assembly of biological and inorganic components is being increasingly developed with the aim of achieving high-quality functional materials exhibiting properties of living species, but entrapped within inorganic solid structures [2–3]. When such assembly processes are carried out at the nanometre scale, the concept of nanoarchitectonics, coined by Japan’s National Institute for Materials Science (NIMS), represents an efficient strategy to develop advanced bionanohybrid and bionanocomposite materials exhibiting specific functionalities. According to recent indications by Ariga and Azzaroni [4], the nanoarchitectonics approach involves the manipulation at the nanometre or molecular level, using chemical reactions, processes of self-assembly and self-organization, as well as modulation under external stimuli. This strategy represents a path of great interest to develop new biohybrid materials harbouring biological functions that can be effectively manipulated through the inorganic components, with potential impact on leading applications within the fields of chemical synthesis and catalysis, energy, environment, and biomedicine.

Examples of bionanohybrids include the bottom-up fabrication of bionanocomposites that display biomimetic and bioinspired characteristics, derived from their biological components (e.g., polysaccharides, proteins, nucleic acids, enzymes and viruses, etc.) and the inorganic network (e.g., silica and silicates, clay minerals and phosphates) [5–8]. More complex biohybrid systems studied during the past decades were based on the encapsulation of enzymes and yeast spores within silica gels for the design of biosensors and bioreactors [9–12].

Biological entities can be considered themselves excellent examples of self-assembly occurring in nature [13]. Based on these complex architectures at the nanometre level, the possibility of artificially designing and building new nanostructured biohybrid materials with functions superior to their separate inorganic and bioinorganic counterparts has become possible.

The entrapment of unicellular microorganisms, such as bacteria, by inorganic components generated by sol–gel processes from silicon alkoxides is an illustrative example of biohybrids that display the extraordinary functionality inherent to the assembled living components. For instance, bacteria such as *Escherichia coli* maintain their metabolic activity entrapped in silica-based inorganic matrices, being viable after one month of encapsulation [12,14–17]. This type of immobilization was also carried out using silica gels and other inorganic systems for the encapsulation of unicellular algae (microalgae) to develop active biohybrid materials that show promising properties for utilization in photobioreactors and biosensor devices [18–21]. According to Fakhrullin et al. [22], cellular nanoencapsulation can be nowadays considered as a promising way to develop more complex systems based on nanoshell assemblies using inorganic nanomaterials as shell components [22–25]. All these biotechnological and multidisciplinary approaches are concerned with cell surface engineering for efficient encapsulation using divers nanomaterials. However, a recent review article published by Homburg and Patel [26] showed the necessity to solve various issues regarding the replacement of toxic precursor components and by-products by non-toxic substances in order to improve viability and/or growth of the entrapped cells. In fact, new organic, inorganic, and hybrid materials for cell entrapment need to be optimised regarding characteristics such as stiffness and abrasion resistance to help the growth of the entrapped cells. In addition, control of the porosity of the involved inorganic materials is key to control efficiently the exchange of metabolites and nutrients with the surrounding environment.

In this context, the present contribution will focus on the application of nanoarchitectonics to the assembly of biological systems based on unicellular microorganisms with inorganic silica and silicate-based substrates. The innovative approach of this work is mainly the comparison of two types of inorganic systems for the immobilization of cyanobacteria and yeast cells. On the one hand, sepiolite clay mineral was used for the immobilization of these microorganisms taking into account that this natural microfibrous Mg silicate presents a wide range of porosity, the ability to generate very viscous stable dispersions, and the capability to form biocompatible nanocomposites by assembly to diverse biopolymers [5,27–28] that can be of crucial advantage for entrapment and viability of the cells. Initial entrapping trials using sepiolite clay derivatives have yielded encouraging results. Hence, this approach has been considered here [29]. On the other hand, the use of yolk–silica shell (YS) microstructures formed by soft template synthesis was explored [30] to encapsulate living cells with a highly porous SiO_2_ network aiming to introduce a small interstitial space between the microorganisms and the silica matrix. The latter strategy intends to confer a shell structure to the unicellular organisms to improve their inherent properties and functions. In fact, the so-called “cellular shellization” has been proposed to obtain a cell surface allowing for applications in advanced technologies and biomedicine including cell delivery and cell therapy for cancer treatment [31].

## Results and Discussion

### Sepiolite–biopolymer microalgal biohybrids

Sepiolite, a microfibrous hydrated magnesium silicate with the formula Si_12_O_30_Mg_8_(OH,F)_4_(H_2_O)_4_·8H_2_O [32–34], shows interesting surface properties and high viscosity [27,33–35]. These properties make it very attractive for various applications [34,36–37] including its controlled modification by assembling diverse components from biological origin [27–28,38–40]. According to the World Health Organization, this natural silicate is classified with “inadequate evidence” of toxicity and carcinogenicity and can be considered biocompatible, which allows its use in fields related to health and food, such as pharmaceuticals, biomedicine, and animal feed [34]. The clay modification with nanometre-scale biopolymers leads to bionanocomposite materials, which are considered as promising nanoarchitectures towards more complex biohybrid materials, such as for the entrapping of microorganisms investigated here. In this context, previous exploratory works in our laboratory showed interesting results regarding the use of sepiolite-based bionanocomposites as alternative to silica-based materials for the entrapment of microalgal species such as *Chlorella vulgaris* and *Anabaena PCC7120* allowing for more than two months of viability [38].

**Figure 1:**
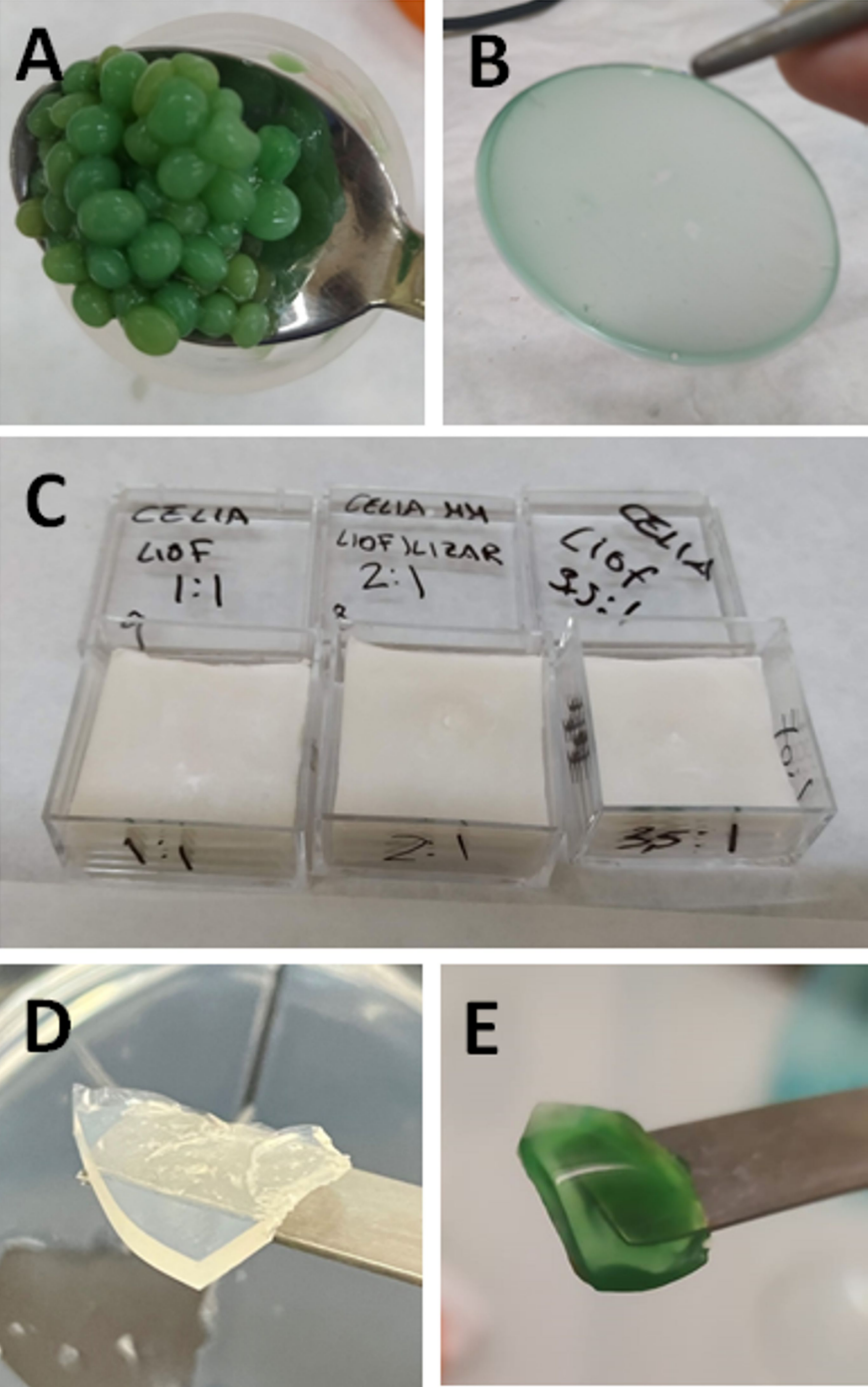
Macroscopic view of prepared biohybrid materials. (A–C) Sepiolite–biopolymer nanocomposites entrapping cyanobacteria (*Synechococcus elongatus PCC 7942*) in diverse conformations. (A) Sepiolite–alginate beads, (B) sepiolite–chitosan thin film, and (C) sepiolite–chitosan foams without cells. (D, E) Silica-based nanocomposites. (D) G57-4-type silica gel without cells and (E) the same type of silica gel with entrapped cyanobacteria cells.

In the present work, various bionanocomposites based on the combination of alginate and/or chitosan and sepiolite have been prepared and processed in different conformations to be tested as immobilization matrices of microalgae. Sepiolite–alginate beads, sepiolite–chitosan/alginate thin films, and sepiolite–chitosan foams were produced (Figure 1). Sepiolite and biopolymer concentration, synthesis temperature, and microorganism concentration were modified to study their influence on the characteristics of the final materials. Thus, an optimised protocol yielding biocompatibility and physical stability, as well as suitable transparency to allow for the viability of cyanobacteria was found.

Previous results on algae biohybrid systems based on chitosan biocomposites have demonstrated great biocompatibility, allowing the cells to survive over long periods of time [38]. However, in the present study using chitosan-based biohybrids, we have observed that the cells did not stay alive for long. The current results point out that chitosan may exhibit antibacterial activity, affecting prokaryotic organisms, such as the cyanobacteria used in this work, more strongly. Yet, it is still an appealing alternative for microalgae or other eukaryotic microorganisms. In the present case, it has been also observed that the prepared chitosan-based foams easily disaggregate in aqueous media, making the infiltration of cells within the foam pores impossible. Alternative systems involving alginate-based biohybrids show that the resulting beads were prone to contaminations, as it can be observed from viability studies (Supporting Information File 1, Figure S2). Additionally, these sepiolite–alginate biohybrid beads strongly limit the diffusion of metabolites, as we determined by means of diffusion studies using Congo red and crystal violet dyes (Supporting Information File 1, Figure S1). It can be concluded that the developed sepiolite–biopolymer nanostructured materials exhibit interesting properties. However, it will be necessary to find alternative protocols to produce substrates exhibiting the properties required for a successful encapsulation of living cells.

### Silica-based microorganism biohybrids

The above-mentioned results regarding the use of clay-based bionanocomposites to immobilize microorganisms indicate strong limitations to an effective encapsulation. Hence, we explored the use of silica gel-based systems considering the good results previously published on the encapsulation of microorganisms using silica-based chemistry [1–2]. Thus, silica gels with low sodium content were prepared by adapting the protocol previously described by Rooke and co-workers [41]. The synthesis conditions were optimised regarding concentration of sodium silicate precursor, temperature, and content of silica nanoparticles (LUDOX^®^ TMA), using a combinatorial exploration of the different synthesis parameters (Table 1). The subsequent qualitative evaluation of the synthesised gels regarding strength, opacity, and stability in cyanobacteria and yeast growth media (BG-11 and apple juice, respectively) after 24 h showed that the optimal conditions were the ones used to produce the G57-4 gel (5% sodium silicate, 7.5% silica nanoparticles, and 4 °C). A possible explanation could be that in the preparation of this gel, a medium concentration of silicate was used, which reacts sufficiently slowly at 4 °C to allow for bonding of the silica nanoparticles, generating a network of high porosity. Visually, the produced G57-4 silica gel material appears to be a rigid, semitransparent, lightly coloured material. Diffusional tests with an identically synthesised silica gel (G57-4 conditions) prepared in the presence of Congo red dye indicated a higher diffusional limit than that of the formerly studied sepiolite–alginate biohybrid systems (Supporting Information File 1, Figure S1).

**Table 1:** Synthesis parameters varied during the optimization of the silica gel preparation.

Sample label	Sodium silicate concentration (wt %)	Silica nanoparticles content (wt %)	Sodium silicate initial temperature (°C)

G35-25	3.0	5.0	25
G37-25	3.0	7.5	25
G35-4	3.0	5.0	4
G37-4	3.0	7.5	4
G55-25	5.0	5.0	25
G57-25	5.0	7.5	25
G55-4	5.0	5.0	4
G57-4	5.0	7.5	4
G75-25	7.5	5.0	25
G77-25	7.5	7.5	25
G75-4	7.5	5.0	4
G77-4	7.5	7.5	4

The optimization study also showed that the use of a sodium silicate concentration of 7.5% may cause an increase in the diffusional limitation, reducing the exchange of compounds necessary for the biological activity of the encapsulated organisms. The reduced gel strength can be compensated with the addition of silica nanoparticles (LUDOX^®^ TMA), for which we have observed a reinforcement of the gel structure without increasing the diffusional limitations of the material. The G57-4 material yields a good balance between robustness, low diffusional limitation to soluble compounds, and transparency. Although the synthesis procedure employed in this work comprised the gel formation within prism-shaped containers, our protocol allows one to create these materials in almost any desired shape and structure. Using custom-shaped moulds, the gel precursor could be cast and gelled as thin films or hollow tubular monoliths with thin walls, improving the interaction between the encapsulated cells and a liquid medium in which the material could be placed.

In addition to the optimisation of the sol–gel synthesis, the conditions for yolk–shell preparation have been also optimised. The protocol reported by Wang and co-workers [25] has been adapted as described in the Experimental section. The optima of LUDOX^®^ TMA concentration and protamine incubation time have been found to be 1 mg·mL^−1^ and 20 min, respectively. Once the optimal synthesis conditions had been determined, the preparation of the G57-4 silica gel substrate was used to produce diverse biohybrid systems incorporating living cyanobacterial or yeast cells, as well as pre-synthesised yolk–shell bionanobybrids. In all cases, the microstructural features of the resulting biohybrid systems were studied by means of optical and electron microscopy (SEM and FE-SEM). Both techniques allowed us to study in detail the cellular arrangement of the microorganisms and their interaction with the inorganic matrix system.

**Figure 2:**
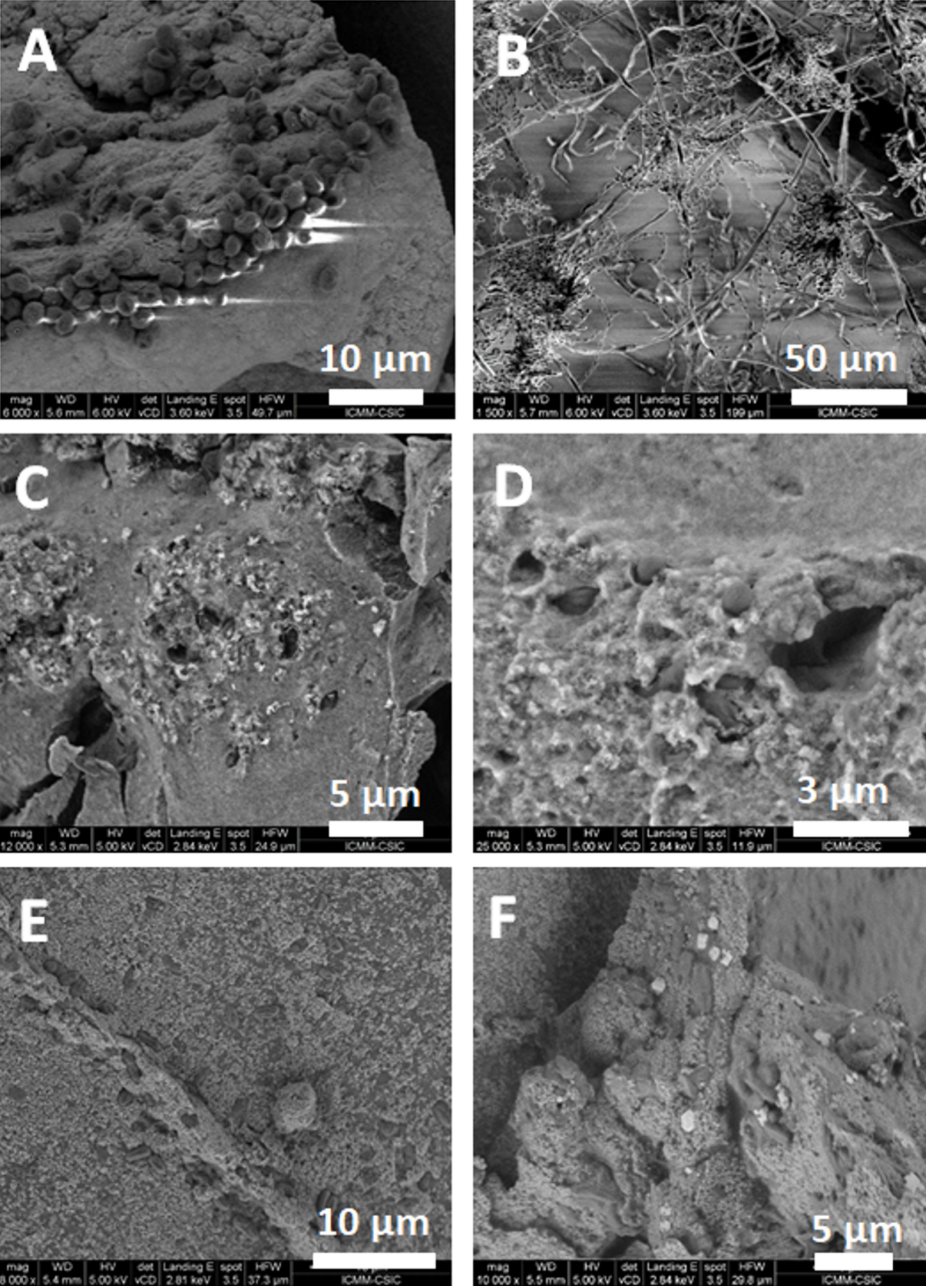
FE-SEM images of silica gel substrates incorporating cells. G57-4 silica gel system with (A) cyanobacteria and (B) yeast. (C, D) SG-YS system with encapsulated cyanobacteria. Cyanobacterial cells encapsulated in yolk–shell structures incubated with protamine for (E) 20 min and (F) 90 min.

FE-SEM microscopy images of the different gel encapsulation systems are shown in Figure 2, confirming the presence of embedded cells or yolk–shell structures (i.e., silica gel with embedded yolk–shell structures (SG-YS)).

Regarding the silica gel biohybrid systems, the cyanobacteria encapsulated in the G57-4 silica gel tend to form aggregates of several tens of cells, where each cell displays a similar size and morphology to cells grown in liquid culture. However, some cyanobacterial cells present a slightly reduced size and a more spherical morphology when compared to their common rod-shaped appearance. Although this change in morphology is correlated with stress adaption, none of the cells display osmotic stress signals, preserving a healthy aspect that can be found when cells are cultured in standard growth media under high CO_2_ levels [17]. In contrast, as displayed in Figure 2B, yeasts encapsulated in the G57-4 silica gel display a completely different morphology. This image focusses on a “hollow region” of yeast cells encapsulated into the silica gel substrate. In Figure 2B, pseudohyphal growth is clearly observed, yielding long filaments of associated cells that expand as a three-dimensional network within the matrix. In this hollow region within the silica matrix, yeast cells display a completely different morphology from that in common suspension cultures. The observed pseudohyphal growth corresponds to morphological changes that are usually correlated to stress for growing yeast cells [16]. Thus, the pseudohyphal growth could be likely caused by the stress imposed by the direct contact with the silica matrix. Cells in interaction with, but not strongly confined by, the matrix quickly start to divide and to proliferate in pseudofilamentous structures [42] that tend to colonize the free space present in the highly porous regions of the silica gel matrix. This behaviour is highly interesting, since it could be exploited in further studies where post-synthesis pseudohyphal growth of yeast cells can generate an intricate tridimensional network of living cells, providing higher mechanical stability and biological robustness, and even an improved transport of nutrients and metabolites across the material.

The FE-SEM images in Figure 2C and Figure 2D show the same microorganism cells but previously encapsulated in yolk–shell microstructures. They are arranged differently from those immobilized freely in the silica gel substrate. In the systems with cells embedded without a previous yolk encapsulation, the cells tend to be more aggregated and loosely attached to the matrix surface. In contrast, the gels containing yolk–shell encapsulated cells present a more homogenous distribution of cells in the matrix. In addition, the cells are more spatially confined in deeper and smaller cavities within the silica matrix. This means that when microorganisms are incorporated as yolk–shell particles, the integration in the silica gel matrix is more homogenous. In addition, in Figure 2D a “hollow space” between the cyanobacteria and the silica matrix is easily observable. This indicates that the cyanobacteria encapsulated in yolk–shell microstructures, instead of being directly attached to an inorganic surface, are floating separated by a space between cell wall and silica shell. This gap is crucial to alleviate the previously observed stress adaptations in cell morphology. Figure 2E and Figure 2F show yolk–shell structures with encapsulated cyanobacteria, where the cyanobacterial cells present their characteristic elongated shape, instead of the more spherical appearance in Figure 2A (direct silica gel encapsulation). This result points out that the usage of yolk–shell structures as a partial replacement of silica nanoparticles during silica gel synthesis leads to a more homogenous distribution of cells and a tighter spatial confinement of the cells within the material. Furthermore, the reduced contact between cells and the inorganic matrix leads to a more biocompatible local environment for individual cells, while providing a more homogeneous and better encapsulation system.

Additionally, in Figure 2E a detail of an isolated yolk–shell structure and an agglomeration of several cyanobacteria encapsulated in yolk–shell microstructures are shown. This image shows how single cells or groups of a few of them can be confined into a self-assembled silica nanoparticle shell in which there is also a small hollow region between the cells and the silica particles. Nonetheless, an excessive presence of silica nanoparticles that has not been self-assembled around the cells is also clearly observable. In Figure 2F one can see how the size of the cells has shrunk compared to other encapsulation systems, demonstrating that when protamine incubation times are too long, the yolk–shell encapsulation cannot proceed properly. According to these results, we have established an optimal protamine incubation time of 20 min (Figure 2E). Although it is appreciable that properly shaped yolk–shell microstructural arrangements have been formed, there is also a lot of smaller particles with a more granular texture surrounding them. These rough particles are probably cyanobacteria directly coated with silica nanoparticles with a lot of smaller silica particles scattered around them. This observation indicates that the yolk–shell synthesis protocol could be further optimised. One improvement for further studies would be to reduce the quantity of silica nanoparticles in the yolk–shell synthesis.

**Figure 3:**
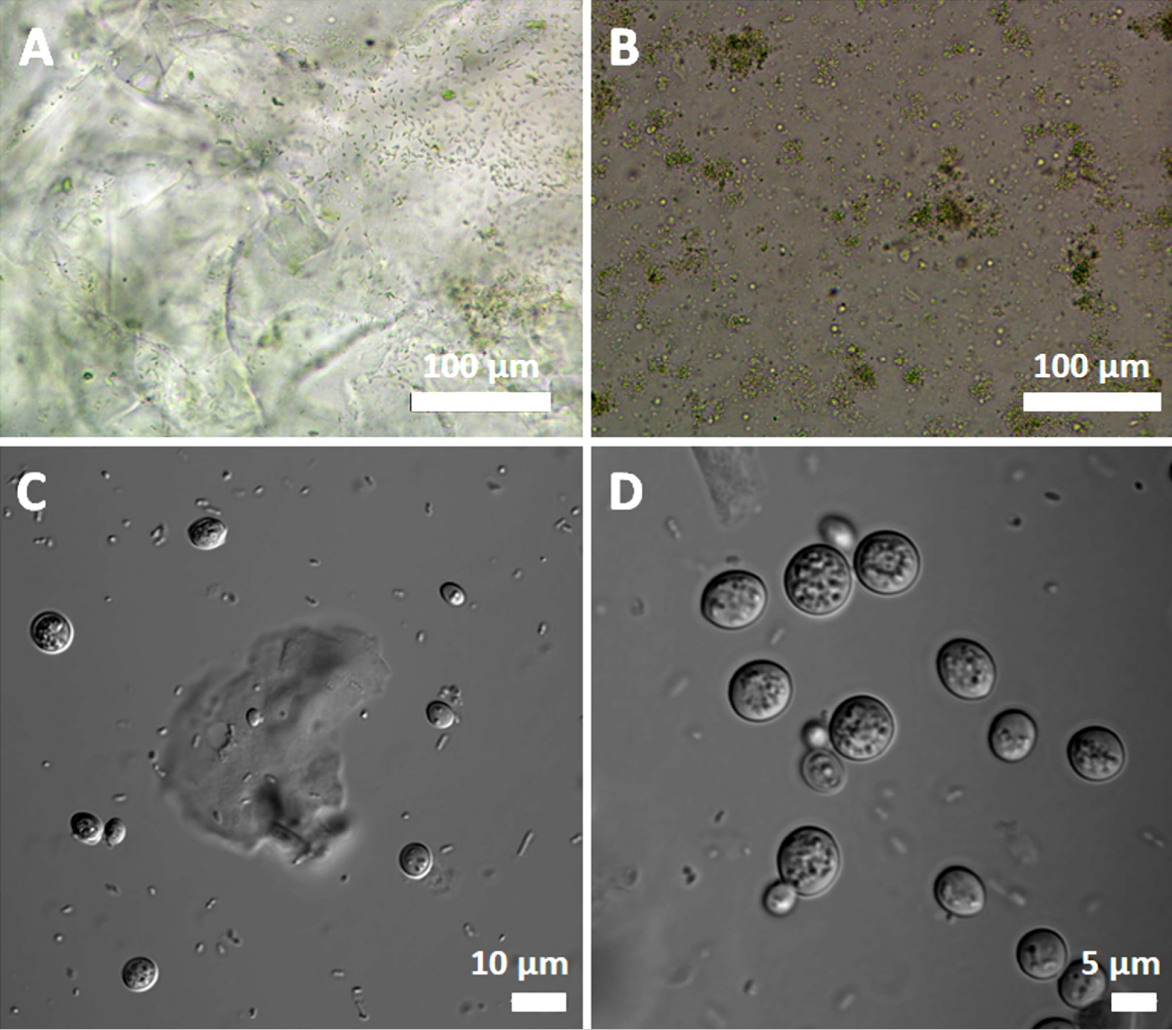
Optical microscopy images. (A) G57-4 silica gel matrix incorporating free cyanobacteria and (B) cyanobacteria embedded within yolk–shells microstructures. (C, D) Bright-field images of yeast cells encapsulated as yolk–shell nanostructures.

Optical microscopy provides a close-to-bulk overview of the material, allowing us to study the cell distribution and homogeneity of the biohybrid materials. Figure 3 displays optical microscopy images of both cyanobacteria and yeast cells.

In the G57-4 material with cyanobacteria, the cells tend to aggregate around gel particles (Figure 3A). When cyanobacteria are encapsulated as yolk–shell structures, the microorganisms achieve a more homogeneous dispersion (Figure 3B). It is worth noting that this is not observed when yeasts are embedded within the material. Figure 3C and Figure 3D show that several yeast cells are embedded in the transparent silica matrix, featuring a visible interface resulting from the shellization process.

**Figure 4:**
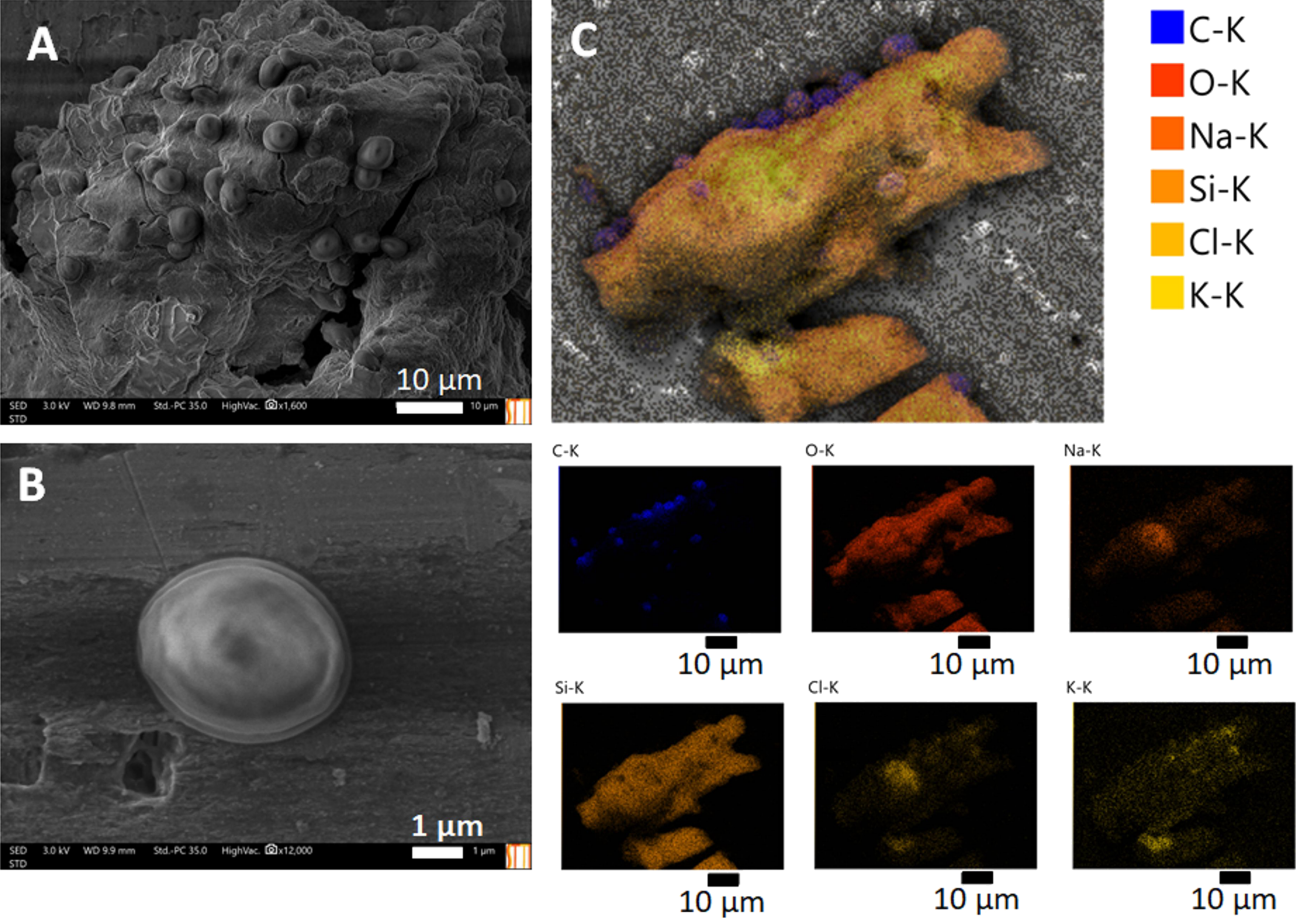
(A, B) Images of the biohybrid prepared from yeast embedded in the silica gel matrix G57-4 obtained recorded with an environmental SEM equipment at different magnifications. (C) Top: Composite image of the silica gel matrix with embedded yeast yolk–shell structures obtained by superimposing the various images of the elements distribution mapping (smaller pictures at the bottom).

The G57-4 silica gel materials with embedded yeasts were studied in further detail using the environmental SEM/EDS equipment described below. SEM/EDS analysis provided detailed information about the structural features of yeast cells embedded in the silica matrix. Figure 4 displays the most relevant findings of this imaging study.

Figure 4A shows that the yeast cells are found in specific points of the matrix, mainly arranged in groups. This distribution may be caused by the active division of the cells within an enclosed cavity. Also, because of the quick gelation of the materials it is likely that a fully homogeneous dispersion of the cells in the material has not been achieved. The arrangement adopted by the cells has a considerable resemblance to the distribution that is found in a liquid culture. This herd-like arrangement could be beneficial, since the cells are organized in a fashion that resembles their normal growth conditions, facilitating their survival and metabolism maintenance.

In contrast to the FE-SEM observations of the yeast–G57-4 nanohybrid system (Figure 2B), isolated yeast cells can be observed. Only in certain regions, there are some yeast groups growing in a protofilamentous fashion. Interestingly, this type of yeast growth is mostly present around smooth surfaces of the inorganic matrix or in places where there are no cavities where the yeast can be entrapped. Figure 4C shows the ESD map (upper image) composed from the elemental distribution images (bottom) of a yeast–silica gel nanoarchitecture. The embedded yeast cells are not fully integrated within the smaller particles of the silica material but attached to its surface. There are also some silica nanoparticles (LUDOX^®^ TMA) covering the cells in contact with the silica gel fragment. This indicates that for yolk–shell formation, the previous incubation with protamine is crucial for adequate self-assembly of a silica nanoparticle envelope.

To conclude this section and considering the above-mentioned results, our observations indicate that the utilization of silica gels with low sodium content greatly increases the biocompatibility of the matrix, sustaining the biological activity of the encapsulated microorganisms. However, direct contact with the inorganic silica matrix and probably the presence of residual reagents still generate stress for the encapsulated cells, which could trigger physiological adaptions to survive within the nanostructured material. Nonetheless, the results obtained with yolk–shell nanoarchitectonics not only indicates that yolk–shell structures could be easily synthesised and used for protecting delicate biological entities, but also that yolk–shell microstructures could be further used as a material itself for assembling more complex biohybrid systems. Our results point out that silica gels containing previously encapsulated cells into yolk–shell structures feature a more continuous silica matrix with deeper cavities in which single cells are entrapped.

### Viability studies

Viability studies have been carried out to analyse the suitability of the encapsulation by studying the performance of each encapsulation system and how well the desired characteristics are achieved. The viability characterisation has been performed semiqualitatively, and the study has been divided in two main areas, namely physical-structural robustness (“macroscopic appearance” and overall “stability” in growth media) and biological viability (“gas release” as a measurement of active metabolism and “cell leakage” measuring the encapsulation strength).

Physical-structural robustness focuses on the evolution of physical appearance and mechanical resistance of the bulk material with time. Macroscopic appearance corresponds to a brief description of the material aspect, while stability corresponds to the observed overall resistance of the material to mechanical strain. Biological viability focuses on the survival state of the encapsulated biological entities. Both yeast and cyanobacteria release gases as products of their metabolism (CO_2_ and O_2_, respectively). Hence, their metabolic activity can be tracked in terms of the gas release, observable as bubbles at the material’s surface. Thus, the gas release is a good indicator of the overall cell viability within the encapsulation system. In addition, the undesired leakage of cells to the surrounding medium can be studied by the presence of an increasing number of cells freely growing in it. The measurement of the absorbance of the culture medium is used to track the number of free cells that are reproducing outside the encapsulation system. For the physical-structural robustness, a small description is indicated, where for the sake of simplicity, the symbol “=” indicates that the observed behaviour corresponds to the one documented on the previous day. In the case of biological viability, the symbol “+” is used on a scale from one to three to describe semiquantitatively the extent of the observed behaviour. Likewise, the symbol “−” indicates the absence of gas release or increase in the absorbance of the growth medium when compared to the previous day. Consequently, a “−“ in “cell leakage” indicates that the absorbance of the growth medium did not change with respect to the previous day, that is, there was no cell proliferation, and the escape of cells from the matrix was negligible. In the case of neat yolk–shell encapsulated cells, the cell leakage has been evaluated mixing the particles in the tube and observing an increase in the absorbance of the supernatant above the sedimented particles. This way, a “−“ sign indicates no appreciable cell proliferation in the growth medium besides the cells already encapsulated in the yolk–shell microstructures.

All these critical features have been studied and compared among the different systems over a course of ten days from the synthesis day, and the results are summarized in Table 2. The key for results interpretation is described in greater detail in the Experimental section.

**Table 2:** Viability studies of cyanobacteria and yeast incorporated into silica-based encapsulation systems.

Day		Macroscopic appearance	Stability and physical properties	Gas release	Cell leakage

1	SG	semitransparent; strong and elastic gel chunks; hard jelly-like consistency	material does not swell; consistent gel; insoluble	−	−
	YS	green (cyanobacteria) or beige (yeast) liquid; homogeneous suspension	mechanical properties cannot be tested. sedimentation after 10 min	+ in the first hours	−
	SG-YS	semitransparent gel chunks; stiffer and more vitreous look compared to SG only	insoluble, well differentiable chunks; more rigid than SG	++ big bubbles	−

3	SG	= to previous day; growth media look more greenish/beige	no change	**+** specially with yeasts	+
	YS	greenish/beige slurry sedimented at the bottom	quick sedimentation after less than 10 min	++	−
	SG-YS	no change	no change	++	−

5	SG	gel chunks are paler; growth media become more greenish/beige	big bubbles are visible outside the chunks; gel feels softer and swollen	**+++** (yeast); **+** (cyanobacteria)	++
	YS	no change	small bubbles are present on top of the sediment particles; no change in sedimentation time	++	+
	SG-YS	no change in gel or growth media appearance	presence of bubbles, but no change in material stability	+	−

7	SG	gel chunks are bigger; supernatant acquires more intense colour because of cell leakage	gel feels softer and tends to break in fragments even under soft shaking	++	=
	YS	no significant changes; less bubbles are observed	no significant changes	+	+
	SG-YS	gel chunks look glassier; gel colour is more intense (less transparency)	no significant changes	++	−

10	SG	small gel chunks are floating within the green/beige growth media	gel fragments easily, even under mild shaking; chunks are softer and easy to break	+	+++
	YS	no significant changes; bubbles are formed within the green/beige slurry	after sedimentation, supernatant has a faint colour (little leakage)	+	+
	SG-YS	big bubbles covering the gel; growth media look slightly more turbid	chunks are covered in bubbles and are a bit softer but do not break easily	+++	+

Figure S2 (Supporting Information File 1) shows an image of all materials tested at different points in time. According to the observed results, yolk–shell microstructures embedded within a silica gel matrix appear as the most suitable candidates for robust, easy-to-handle biohybrid materials to retain the bioactivity of the encapsulated microorganisms. It should be considered that, although the viability in sepiolite–biopolymer materials has been studied, the poor performance in terms of desired features for a biophotocatalytic application (poor transparency and reduced long-term biological or physical stability) has led us to discard them from a more intensive study.

### Ethanol production of encapsulated yeast

The biological production of ethanol from the biohybrid yeast–silica-based materials has been qualitatively studied to assess the biocatalytic potential of the system. One of the key aims of our research is to study whether an encapsulation system can be used to easily recover secreted metabolites of interest from outside the silica matrix. Thus, the biohybrid material could be used as biocatalyst. The formerly described materials exhibit properties that make them suitable for this application. However, because of time limitations to our research, this possibility has not been fully explored yet. Nonetheless, ethanol production of encapsulated *S. cerevisiae* in the formerly described silica-based nanoarchitectonics systems can be used as a way of testing the biocatalytic potential of these systems. Since this yeast species secretes ethanol naturally as a product of its native metabolism, ethanol production can be measured. Following the method described in the Experimental section, we have evaluated the ethanol production of encapsulated cells and compared it to the production in a standard suspension culture. The high variability in the densitometric quantification of the ethanol concentration led us to consider these results only as a tentative experiment to evaluate the biocatalytic performance. However, these preliminary findings combined with the observed CO_2_ release from the cultures and the detection of alcohol allowed us to confirm that the encapsulated yeast produced ethanol likely faster and in higher amounts than free cells (Table 3). This behaviour may indicate that encapsulation indeed promotes a metabolic shift towards metabolite production rather than biomass formation. However, it is worth noting that after five days of growth, the ethanol concentration was probably sufficiently high to render the encapsulated yeast inactive or even kill it. This fact points out that further studies of the biocatalytic potential of encapsulated microorganisms must be conducted. Despite the fact that encapsulation promoted higher productivities, its protective effect against toxic metabolic products is limited, indicating that product removal must be considered as a way to retain the material’s bioactivity.

**Table 3:** Ethanol production experiments for yeast encapsulated within silica gel biohybrid materials. ρ denotes the density of the culture supernatant measured at 20 °C. The ethanol concentration has been estimated using reference data on ethanol–water mixtures [39]. NM indicates that the density could not be reliably measured.

Free cell cultures				Immobilized **y**east **c**ultures			
	
day	ρ (g·mL^−1^)	% EtOH	CO_2_ release	day	ρ (g·mL^−1^)	% EtOH	CO_2_ release

0	1.038	0	yes	0	1.038	0	yes
6	0.987	7.1	yes	5	0.979	12.9	yes
14	NM	NM	yes	14	NM	NM	no

## Conclusion

**Figure 5:**
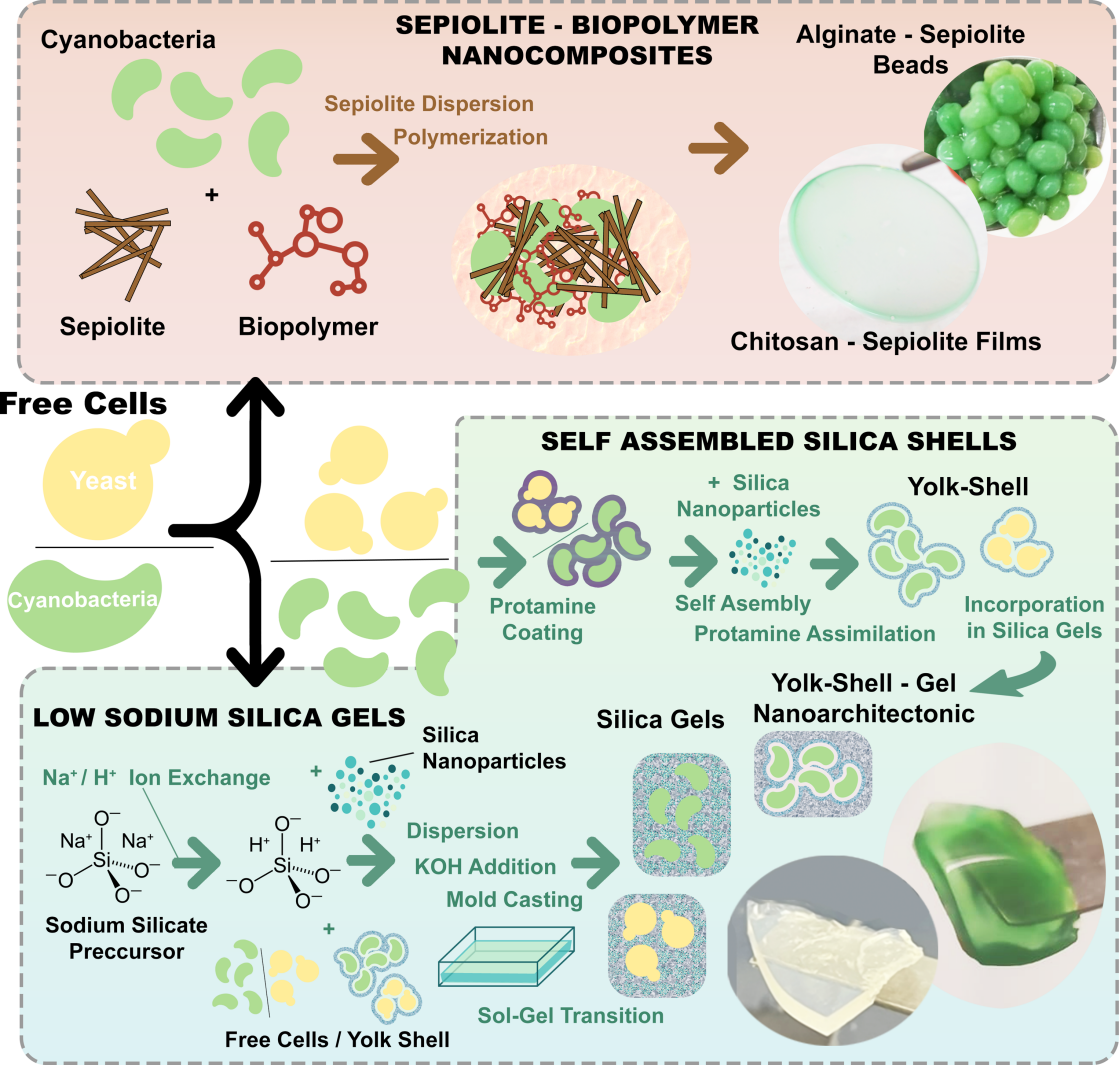
Schematic representation of the nanoarchitectonics approaches applied in this work for the immobilization of living microorganisms in silica-based systems.

The present contribution describes the application of the nanoarchitectonics concept to contribute to the development of new bio-inorganic systems based on the assembly of living unicellular microorganisms with a clay mineral such as sepiolite and other silica-based porous materials as summarized in Figure 5. Silicate-based bionanocomposites derived from fibrous sepiolite silicate assembled with alginate and chitosan biopolymers have been tested as support of cyanobacteria and compared regarding the survival of cyanobacteria. The best system synthesised here was based on sepiolite modified with alginate and conformed as millimetre-sized beads after biopolymer reticulation by CaCl_2_ treatment. Yet, cell entrapment using silica-based systems such as yolk–shell silica microstructures formed by soft template synthesis appeared as a more efficient way to encapsulate living cells. In fact, cellular shellization with a highly porous SiO_2_ network and a small interstitial space between the cellular microorganisms and the silica matrix turned out to be a proficient cell surface nanoarchitectonics strategy. Colloidal synthesis of materials using silica gels with low sodium content as an alternative to sepiolite–biopolymer nanocomposite materials for microorganism encapsulation increased the biocompatibility of the matrix, sustaining the biological activity of the encapsulated microorganisms. Furthermore, a protocol combining self-assembly of silica nanoparticles and sol–gel chemistry has been developed here. It allows one to fine-tune the nanoarchitecture, leading to novel silica-based biohybrid systems that are able to retain the biological activity of the encapsulated cells while providing protection from external hazards and ensure adequate exchange of nutrients and metabolites with the external medium. The materials synthesised here have shown excellent biocompatibility with both cyanobacteria and yeast cells, retaining metabolic activity and extending biological viability. This feature, in combination with transparency, easy tuning, and low energy requirements of the bottom-up synthesis, opens the way for the development of novel biohybrid systems with a wide range of applications, from biological preservation of living cells to the development of novel whole-cell bioinorganic catalytic materials. Nonetheless, further research in biocatalytic applications of these silica-based nanoarchitectures and optimization of the synthesis procedures is still required.

## Experimental

### Materials and methods

Sepiolite from Vicálvaro-Vallecas deposits (Madrid, Spain) was provided by TOLSA, S.A. as Pangel S9 rheological grade commercial product (>95% purity). A SEM image of this sepiolite sample showing its characteristic microfibrous morphology is provided in Figure S3 (Supporting Information File 1). Chemical reagents such as LUDOX^®^ TMA colloidal silica, potassium hydroxide pellets ≥85%, sodium silicate 37%, sodium alginate of medium viscosity, CaCl_2_·2H_2_O (99%), medium molecular weight chitosan, and BG-11 culture medium have been purchased from Sigma-Aldrich. Protamine sulfate and PBS buffer (biochemistry grade) have been supplied by Thermo Scientific. Commercial food-grade apple juice (Granini) without additives has been used for yeast cultivation. Two different unicellular microorganisms have been used. The wild-type cyanobacteria *Synechococcus elongatus* PCC7942 was used as a representative model of phototrophic prokaryotic microorganisms, and a wild-type *Saccharomyces cerevisiae* strain was selected as a model of heterotrophic eukaryotic microorganisms. The cyanobacterial strain has been obtained from stock cultures of a previous research project, developed in the summer of 2021 for the international Genetically Engineered Machine (iGEM) competition by MADRID_UCM 2021 iGEM team [43]. All procedures involving yeast cells have been performed with a commercial *Saccharomyces cerevisiae* species. Since the aim of this work is not focusing on specific genetic or metabolic features of the employed organisms, the yeast cells have been obtained from a commercial dry-stabilized baking yeast from a local supermarket (Levanova, fresh yeast).

All employed materials and equipment have been sterilized by heating to 120 °C for 25 min and subsequent cool down to room temperature within the autoclave chamber. All culture handling operations have been performed within a sterile laminar flow hood, which before and after its usage had been disinfected with alcohol and sterilized with the ultraviolet light attached to the hood.

Biohybrid sepiolite–biopolymer nanocomposites materials have been prepared adapting a protocol developed in-house previously [8]. A sepiolite slurry (10% w/v clay/water) was mixed with a concentrated biopolymer solution under stirring until the formation of a homogeneous system with a final concentration of sepiolite between 2% and 5%. The employed biopolymer solutions were sodium alginate (8% w/v) or chitosan (4% w/v). Depending on the desired material, different final concentrations of sepiolite–biopolymer were assayed. In the case of sepiolite–alginate beads, after homogeneous dispersion, the mixture was introduced into a syringe and slowly introduced dropwise into a calcium bath (CaCl_2_·2H_2_O, 6% w/v) to form small alginate droplets that hardened via crosslinking the alginate chains with the calcium ions present in the bath. The formed beads were left in the calcium bath for 30 min and then recovered by filtration and washed with distilled water to remove excess calcium chloride. In the case of sepiolite–chitosan materials, the homogeneous suspensions were poured into a 7 cm diameter Petri dish until reaching a depth of 5 mm. Then the material was air dried for at least 24 h until a viscous, non-sticky elastic film was formed. For sepiolite–chitosan foams, the same preparation procedure was followed, but the sepiolite–chitosan dispersion was not mixed with biomass and freeze-dried instead of air-dried, obtaining highly porous biohybrid foams. The lyophilization was performed at −54 °C and 0.01 mbar for 24 h in a Cryodos freeze-drier from Telstar.

Silica gel synthesis involved three steps, that is, microorganism cultivation, creation of the acidic silicate precursor, and synthesis of the biohybrid system. The microorganisms to be encapsulated were cultivated and 30 mL of culture medium at an optical density of 0.8–1.0 were taken during the exponential growth phase and then centrifuged for 10 min at 8000 rpm and 24 °C. The supernatant was discarded and the cells were re-suspended in 1 mL of culture medium and used immediately for gel synthesis. In parallel, the gel precursor was prepared from 37% (v/v) commercial sodium silicate, which was ion-exchanged using a custom-made acidic ion exchange column. After ion exchange, the resulting silica precursor was mixed with LUDOX^®^ TMA silica nanoparticles to reach the desired weight percentage (5.0% to 7.0%). Eventually, the concentrated cell biomass (33% to 40% v/v) was then added and the mixture was placed in a 7 cm diameter Petri dish, where it gelled at room temperature after the addition of 0.2 M KOH to achieve pH 7–8. Table 1 gathers the different conditions used for the silica sol–gel syntheses.

The yolk–shell microstructures have been synthesised adapting the protocol first reported by Wang et al. [25]. For the silica gel syntheses, cells were centrifuged and re-suspended in 1/10th of the initial volume of sterile PBS buffer. The pellet was re-suspended by shaking the tube or by slowly pipetting up and down with a wide-open pipette tip. Then, 1000 µL aliquot from the re-suspended cells was used to measure the absorbance at 720 nm (OD720) using a UV–vis spectrophotometer. The OD720 should be 1.3 or in the range of 1.5–1.7 for cyanobacteria and yeasts, respectively. For greater OD values, more sterile PBS was added till reaching the required OD720 value. Subsequently, a 1/50 (v/v) amount of 5% protamine sulfate in sterile PBS was added to the cell suspension. The tube was flicked up and down a couple of times and incubated for 50 min (no agitation was required). After incubation, cells were centrifuged at 2800 rpm for 10 min, the cell pellet being then re-suspended in the same volume formerly used. Then, the pretreated cells were submitted to the yolk–shell self-assembly. First, the necessary amount of silica nanoparticles was added to reach a final concentration of 1 mg/mL in the cell suspension. After adding the silica nanoparticles, the suspension was incubated for 20 min under mild agitation using a rocker shaker at minimum speed. Yolk–shell structures were recovered by centrifugation at 2800 rpm and washed with fresh BG-11 medium or apple juice, when cyanobacteria or yeasts were to be encapsulated, respectively. The same amount of volume used during incubation was used to wash the cells twice with fresh media. Additional information regarding the growth and handling conditions for the employed microorganisms is included in Table S1 of Supporting Information File 1.

The viability experiments consisted of placing a small amount of each material (0.6 to 1 g) within a sterile 14 mL Falcon tube containing 7 mL of sterile growth media. The tubes were kept under mild shaking and illumination during the whole experiment, being observed and opened daily in a sterile environment for gas exchange. On days 1, 3, 5, 7, and 10, the tubes were kept still outside the incubator for 1 h to observe the behaviour of each material.

For the diffusional limitation studies, 100 µL of saturated solution of the dye were mixed with 15 mL of the synthesis mixture and the biohybrid material was prepared following the previously described procedures. Then, 2 g of the material was placed inside a 50 mL Falcon tube with BG11 media and agitated at 180 rpm, sampling 1 mL of liquid every 1, 2, 3, 4, 10, and 30 min for absorbance measurements. The absorbance of Congo red and crystal red was measured at 541 and 400 nm, respectively.

The ethanol production has been studied using single cultures of *S. cerevisiae* either as free cells or as cells immobilized within the silica matrix. Both cultures were kept under the same conditions, taking 2 mL samples and measuring the density of the supernatant after centrifugation of the culture to remove the suspended solids. Density measurements were performed by weighing 1 mL of supernatant on an analytical balance. It also worth noting that the free-cells culture was inoculated with the equivalent amount of biomass immobilized within the silica gel. To perform this calculation, the total optical density of yeast culture used during the silica gel synthesis protocol was considered. The weight of the gel fragment used for production was previously recorded. With this data and the optical density of the concentrated yeast culture used as inoculum, the volume of inoculum for the yeast suspension culture was obtained.

### Characterisation techniques

Optical microscopy was performed using a Nikon Eclipse LV 100 POL polarizing microscope equipped with an Olympus DP12 digital camera. Samples were observed directly without staining. Electron microscopy imaging was conducted using a field-emission scanning electron microscope FEI-NOVA NanoSEM 230 equipped with an Apollo XL silicon drift detector from EDAX-Ametek or using a high-resolution JEOL IT500HR/LA microscope equipped with an energy dispersive X-ray spectroscopy (EDS) detector. In certain studies, samples were cut after immersing the beads, films, or foams in liquid N_2_ to obtain clean cuts and then sputtered with a thin layer of Au using a Leica EM ACE200 sputterer to assure good conductivity. Using a Zeiss LSM 800 microscope, transmitted-light images in bright-field mode were recorded. UV–vis spectroscopic measurements were conducted on a Shimadzu UV-1201 UV–vis spectrophotometer.

## Supporting Information

File 1 Additional experimental data.
