# Supplementary material for: Nanoarchitectonics to entrap living cells in silica-based systems: encapsulations with yolk–shell and sepiolite nanomaterials
Source: Beilstein J Nanotechnol. 2023 Apr 25;14:522–34. doi: 10.3762/bjnano.14.43 (PMC10155620; doi:10.3762/bjnano.14.43)
Supplement: File 1 — Additional experimental data. [file Beilstein_J_Nanotechnol-14-522-s001.pdf]

BEILSTEIN JOURNAL OF NANOTECHNOLOGY

Supporting Information

for

Nanoarchitectonics to entrap living cells in silica-based systems: encapsulations with yolk–shell and sepiolite nanomaterials

Celia Martín-Morales, Jorge Fernández-Méndez, Pilar Aranda and Eduardo Ruiz-Hitzky

Beilstein J. Nanotechnol. **2023,** *14,* 522–534. doi:10.3762/bjnano.14.43

Additional experimental data

License and Terms: This is a supporting information file under the terms of the Creative Commons Attribution License (https://creativecommons.org/licenses/by/4.0). Please note that the reuse, redistribution and reproduction in particular requires that the author(s) and source are credited and that individual graphics may be subject to special legal provisions.
The license is subject to the *Beilstein Journal of Nanotechnology* terms and conditions: (https://www.beilstein-journals.org/bjnano/terms)

Table S1: Growth and handling conditions for the employed microorganisms.

Conditions	Cyanobacteria	Yeasts
culture temperature	23–28 °C	20–30 °C
culture agitation	90–120 rpm	90–120 rpm
culture medium	BG-11	apple juice
centrifugation	3000 rpm, 10 min	8000 rpm, 10 min
gelation time[a]	24–48 h	30–60 h

[a]Without cells, the time reduces to 8–10 h.

Silica-based systems: preparation notes

For silica gels, depending on the synthesis requirements, the gel can be slightly heated to accelerate gelation. Care should be exercised, as the cells should not be exposed to temperatures above 35 °C. Once the gel synthesis is completed, the system placed in sterile material is stored in a refrigerator (3–5 °C) for yeast cells or at room temperature (20 °C) and low light for cyanobacterial cells, keeping it in contact with culture medium (apple juice or BG11, respectively). This medium must be renewed periodically every 7 to 14 days.

Ludox volume calculation necessary for the yolk–shell syntheses:

$$V_{\text{LUDOX TMA}} = V_{\text{LUDOX}}\left(\mu\text{L}\right) = \frac{418}{V_{\text{cell suspension}}\left(\text{mL}\right)} \cdot 1000.$$

Note: Ludox TMA has a density of ρ=1.23 g/mL, and it is a colloidal suspension of 34 wt %. Hence, 1 mL of the stock solution will carry 418 mg of silica nanoparticles. To know the required amount of LUDOX, just divide the cell suspension volume by 418.

Diffusional limitation studies

The diffusional limitation for soluble non-volatile molecules of the different systems has been studied spectrophotometrically, measuring the diffusion of Congo red and crystal violet from the material to the surrounding medium. Figure S1 displays the resulting measured absorbance. Fitting curves are plotted only as a guide to the eye. Natural logarithmic fittings have been used for all the figures, with the exception of the silica gel, for which a linear regression provided a better result. Although the duration of the diffusion experiment did not allow the systems to achieve equilibrium, these results allow one to easily compare how fast the dyes diffused from the nanostructures to the surrounding medium.

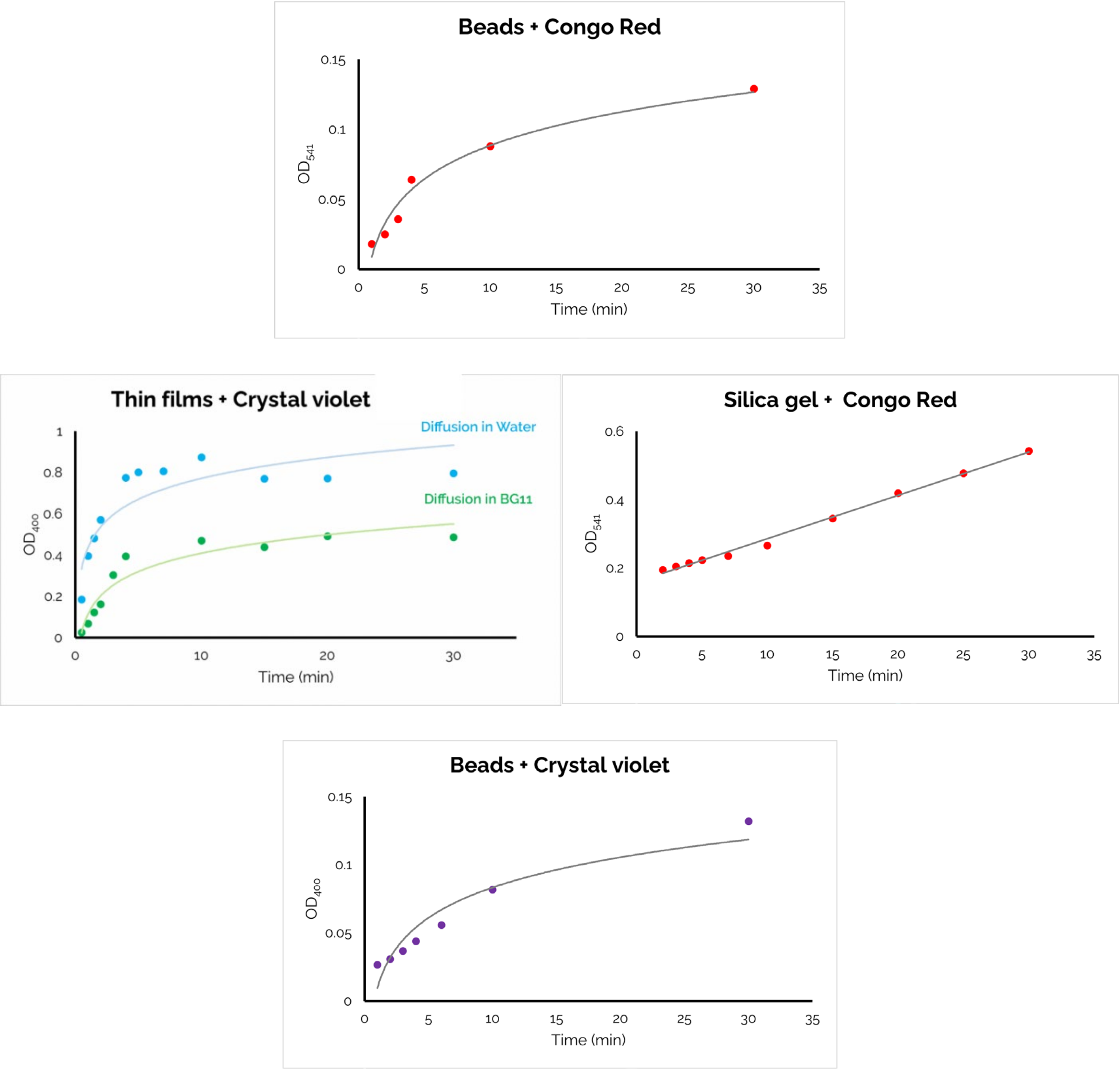

Figure S1: Diffusion of Congo red and crystal violet dyes from different silica-based materials. These materials are sepiolite–alginate beads, sepiolite–alginate thin films, and silica gel G57-4. For the chitosan thin films, diffusion has been studied either in water or BG-11, shown in blue or green, respectively.

Viability studies

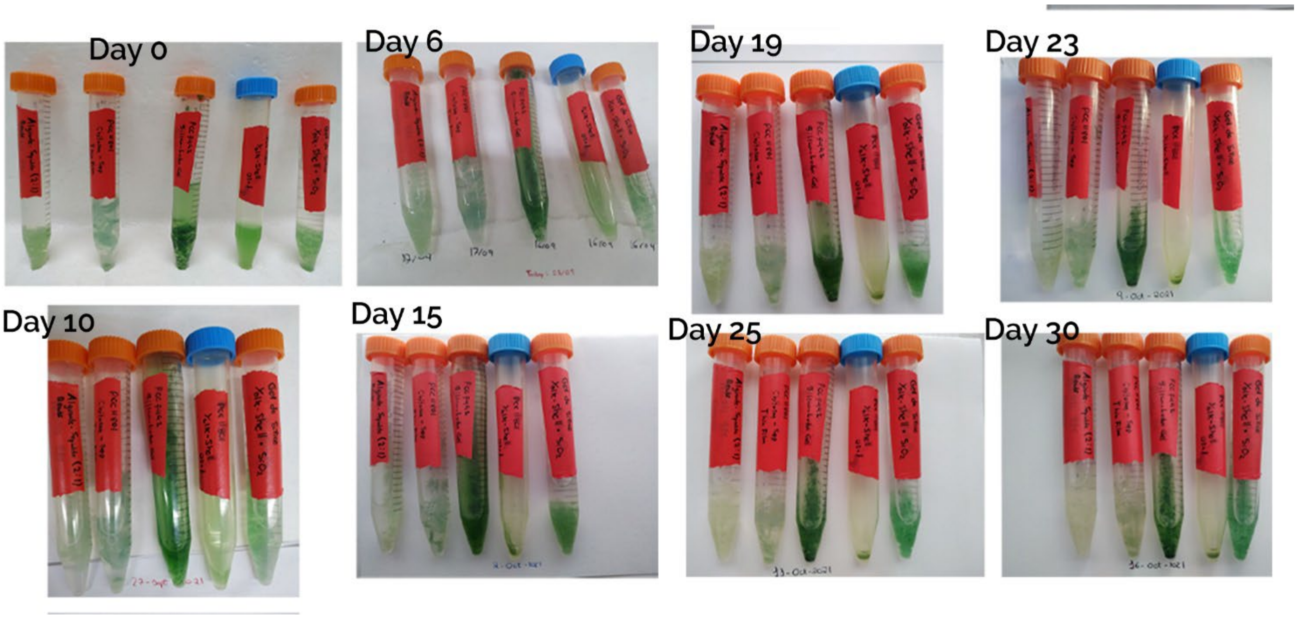

Figure S2: Physical appearance of the materials inside the Falcon tubes during the viability studies. From left to right in each picture: sepiolite–alginate beads, sepiolite–chitosan thin films, silica gel, yolk–shell structures, and yolk–shell structures embedded within a silica gel matrix.

SEM characterisation

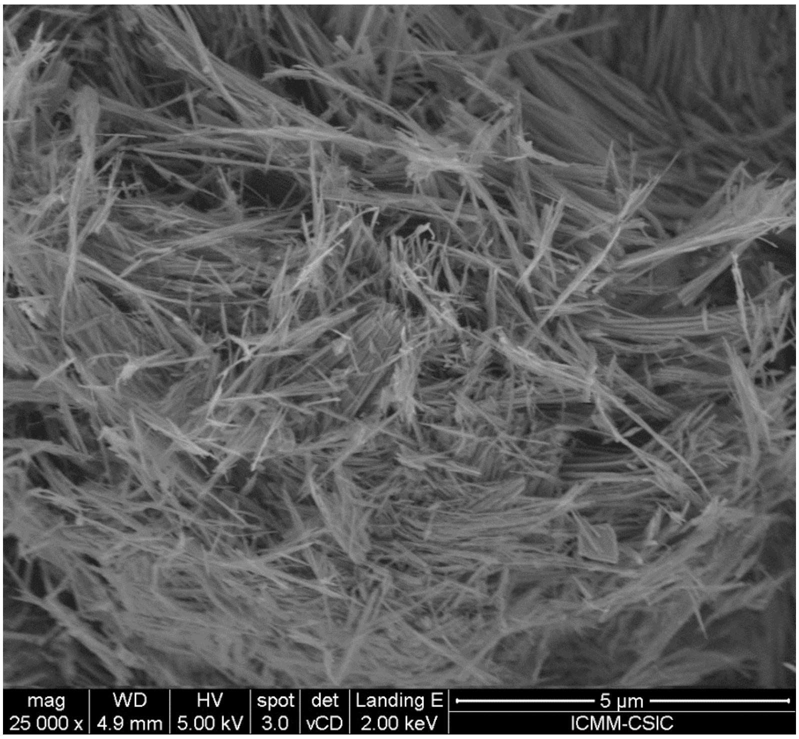

Figure S3: Microstructural SEM characterisation of the sepiolite used in this study.
